# Clinico-Pathological Study of K-ras Mutations in Colorectal Tumors: A Single-Center Retrospective Study of 51 Patients in Madinah, Saudi Arabia

**DOI:** 10.7759/cureus.9978

**Published:** 2020-08-24

**Authors:** Nasser Mulla, Abdulraheem Alshareef, Abdul Rahman Syed, Majid Al-Jahel

**Affiliations:** 1 Department of Internal Medicine, College of Medicine, Taibah University, Madinah, SAU; 2 Department of Medical Laboratories Technology, College of Medicine, Taibah University, Madinah, SAU; 3 Department of Pathology, King Fahad Hospital, Ministry of Health, Madinah, SAU; 4 Department of Oncology, King Fahad Hospital, Ministry of Health, Madinah, SAU

**Keywords:** colorectal cancer, crc, k-ras mutations, madinah, saudi arabia

## Abstract

Background

Colorectal cancer (CRC) is one of the leading types of cancer worldwide and in Saudi Arabia. At the molecular level, CRC is very complicated and requires establishing comprehensive patient stratification models through identification of patients who will benefit or will not benefit from targeted therapy. We retrospectively investigated and analyzed the frequency of Kirsten-ras (K-ras) mutation and its correlation with patients’ characteristics as weel as its association with clinicopathological features (i.e age, gender, clinical stage, anatomical site, histological subtype, degree of histological differentiation and metastatic site) in patients with CRC.

Methods

Medical records and paraffin-embedded tumor samples from 51 patients with histologically proven colorectal adenocarcinoma referred to Madinah center in Saudi Arabia were analyzed for the occurrence of rat sarcoma virus (RAS) mutations.

Results

RAS mutations occurred in 43% of the patients; 91% of these mutations were in K-ras. Seventy-five percent of these K-ras mutations were in codon 12, most commonly p.G12D. Codon 13 mutations occurred in 20% of tumors: all of these were p.G13D (100%). The percentage of K-ras mutations occurrence was higher in young patients (≤50) compared with the older patients (>50) (54.5% and 35%, respectively). Similarly, the percentage of K-ras mutations occurrence was higher in the right-sided tumors compared with the left-sided tumors (57.1% and 32.4%, respectively). Patients’ characteristics and clinicopathological features were not significantly associated with K-ras mutations.

Conclusions

K-ras mutations are common among Saudi patients diagnosed with CRC in Madinah, especially pG12V and pG12D in codon 12. Further investigation would be required to establish correlation of K-ras mutations in larger cohorts.

## Introduction

Colorectal cancer (CRC) is one of the leading types of cancer worldwide [1,2]. In Saudi Arabia, CRC is the most common cancer among males (11.2%) and the third most common cancer among females (8.8%) [3,4]. CRC is very complicated at the molecular level which makes it reasonable to predict that patient’s management is required to be stratified. To establish comprehensive patient stratification models, identification of targetable biomarkers which directs to therapeutic personalization will be crucial [5].

Kirsten-ras (K-ras) is an oncogene that is reported to be activated through mutations in 35% to 45% in patients with CRC [2]. Almost all of these K-ras mutations occur in codons 12 or 13 [6]. K-ras is the most common oncogene in the well-studied rat sarcoma virus (RAS) subfamily proteins because of its significant role in cancer [7]. Wild type or unmutated K-ras protein resides in the GDP-bound state on the plasma membrane in quiescent cells (i.e inactive). However, mutated K-ras will be in continuous stimulation which leads to the recruitment of specific guanine nucleotide exchange factors and significant conformational changes of the K-ras protein. These changes result in constitutive engagement and activation of K-ras effector pathways such as MAPK and PI3K-AKT signaling pathways. Subsequently, mutant K-ras proteins are driving tumor formation and progression [8]. Activated K-ras was shown to be associated with decreased responsiveness to targeted therapies such as anti-epidermal growth factor receptor (EGFR) antibodies in metastatic CRC as well as increased aggressiveness of CRC [9-12]. Therefore, characterizations of K-ras and other oncogenic drivers are increasingly useful to guide prognostication and molecularly stratified treatment options available to patients with CRC [13,14].

In this study, we aimed to investigate retrospectively the frequency of K-ras mutation and analyze its correlation with patients’ characteristics and its association with clinicopathological features (i.e age, gender, clinical stage, anatomical site, histological subtype, degree of histological differentiation and metastatic site) in patients with CRC.

## Materials and methods

Study population

A retrospective study was done at King Fahad Hospital, Madinah, Saudi Arabia. Histopathological and clinical data were obtained from the medical records of 51 patients with histologically proven colorectal adenocarcinoma. Tumor samples were obtained during surgery or biopsies carried out as part of routine care between 2016 and 2019. The histopathology reports were retrieved from the hospital’s laboratory information system and were analyzed for data pertaining to age, gender, site of tumor, histologic type, tumor grade, nodal status and stage. The medical imaging records provided information on metastatic site. The RAS mutation results for all the cases were known, testing was done at Genekor Labs, Greece. All aspects of the study were approved by the local ethics committee.

K-ras and N-ras mutation testing

DNA extraction was carried out from formalin fixed paraffin embedded tissue. 6µ thick sections were taken on positively charged slides from a tumor block selected by the primary reporting histopathologist. These were sent to a referral laboratory (Genekor Lab, Greece). Macrodissection of the tissue was performed on direction of the histopathologist at the referring laboratory by marking the area on the template slide. Tumor cell content (TCC) >75%. DNA was extracted from the sample under investigation (QIAmp DNA FFPE tissue kit, QIAmp DSP DNA Mini kit).

A targeted resequencing assay (Ion AmpliSeq NGS Panel, Thermo Fisher Scientific, Indianapolis, IN) was used for mutation detection in exons 2, 3 and 4 of the K-ras and N-ras genes. Sequencing was carried out using the Next Generation Sequencing Platform Ion Gene Studio S5 Prime System (Thermo Fisher Scientific). The kit was used to detect mutation in p.G12S, p.G12R, p.G12C, p.G12D, p.G12A, p.G12C on Codons 12 and 13 of exon 2. Exon 2 wild type cases were further analyzed for mutations in exons 3 & 4 of K-ras genes and exons 2, 3 & 4 of N-ras genes. The detection limit of the used method was 2%-5% of mutant allelic content, depending on the genomic region analyzed.

Study design

The overall frequency of the K-ras and N-ras mutations including type of mutation and affected codon were tabulated for all the cases with respect to age, gender, tumor site, tumor histologic type, American Joint Committee on Cancer (AJCC) stage and site of metastases. SPSS software (v20) was used to perform the statistical analysis. P <0.05 was considered statistically significant.

## Results

1) Characteristics of patients

Fifty-one patients with CRC were evaluated. The mean age was 60.2 years (range, 28-91). There was no difference in male to female ratio (i.e 1.04:1). In addition, all stages are included in the cohort. Most tumors were left sided (i.e 62.7%). Adenocarcinoma accounts for 94% of the tumors and the site of metastasis was mainly liver. All the other tumor characteristics are shown in Table 1.

**Table 1:** Tumor characteristics of 51 patients with colorectal cancer

	N	%
Age		
≤ 50	11	21.6
>50	40	78.4
Gender		
Male	26	51.0
Female	25	49.0
Stage		
stage I	5	9.8
stage II	13	25.5
stage III	16	31.4
stage IV	17	33.3
Site		
Left	37	72.5
Right	14	27.5
Histology		
Adenocarcinoma	48	94.1
Mucinous carcinoma	3	5.9
Tumor differentiation		
Moderate	43	84.3
Poor	2	3.9
Well	6	11.8
Site of metastasis (n=15)		
Liver	7	46.6
liver, lung	2	13.2
liver, omentum	1	6.7
Lung	1	6.7
lung and liver	1	6.7
mesentry, liver	1	6.7
Omentum	1	6.7
Retroperitoneum	1	6.7

2) Characteristics of K-ras mutations

Mutational analysis revealed RAS mutations in 43.1% (22/51) of CRC patients (Table 2). Most mutations were in K-ras which accounts for 91% (20/22) while the remaining 9% (2/22) were detected in N-ras. In other words, K-ras mutations occurred in 39.2% of the patients (20 out of 51). Seventy-five percent of these K-ras mutations were in codon 12 (15 out of 20), most commonly p.G12D (53% of codon 12 mutations). Codon 13 mutations occurred in 20% of tumors; all of these were p.G13D (100%).

**Table 2:** Characteristics of rat sarcoma virus (RAS) mutations

	N	%
RAS status		
Wild type	29	56.9
Mutant type	22	43.1
Mutant type N=22		
K-ras	20	90.9
N-ras	2	9.1
K-ras (n=20)		
Exon 2	19	95
Exon 4	1	5
K-ras (n=20)		
Codon 12 (p.G12)	15	75
Codon 13 (p.G13D)	4	20
Codon 14 (p.A14)	1	5
K-ras Codon 12 (p.G12) (n=15)		
p.G12A	1	6.7
p.G12C	1	6.7
p.G12D	8	53.3
p.G12S	2	13.3
p.G12V	3	20
N-ras (n=2)		
Exon 2	1	50
Exon 3	1	50
N-ras (n=2)		
p.G12D	1	50
p.Q61L	1	50

3) Correlations between K-ras status (mutation vs wild type) and clinicopathological features

The percentage of K-ras mutations occurrence was higher in young patients (i.e ≤50) compared with the older patients (>50) (54.5% and 35%, respectively). Similarly, the percentage of K-ras mutations occurrence was higher in the right-sided tumors compared with the left-sided tumors (57.1% and 32.4%, respectively) (Table 3). On the other hand, the percentage of K-ras mutations occurrence was comparable between male and female (42.3% vs. 36%) or between stages I to III and stage IV (38.2% vs. 41.2%). There was no statistically significant association between the overall frequency of K-ras mutations and patients’ characteristics and/or clinicopathological features (Table 3).

**Table 3:** Patients’ characteristics and association between clinicopathological features and Kirsten-ras (K-ras) status

	K-ras mutational status No (%)		P value
	Wild type	Mutated	
Age			
≤ 50	5 (45.5)	6 (54.5)	0.24
>50	26 (65)	14 (35)	
Gender			
Male	15 (57.7)	11 (42.3)	0.64
Female	16 (64)	9 (36)	
Stage			
Stages I, II & III	21 (61.8)	13 (38.2)	0.84
Stage IV	10 (58.8)	7 (41.2)	
Site			
Left	25 (67.6)	12 (32.4)	0.11
Right	6 (42.9)	8 (57.1)	
Site of metastasis (n=15)			
Liver	9 (75)	3 (25)	0.17
Non-liver	1 (33.3)	2 (66.7)	

## Discussion

Here, we are reporting the clinicopathological features of K-ras mutations in tumors derived from 51 patients diagnosed with colorectal cancer from 2016 to 2019 in Madinah city (western Saudi Arabia). We have found that 43% of the cohort carries RAS mutations with one-third of these mutations localized within codon 12 of K-ras. Characterizing the clinicopathological features of K-ras mutations in CRCs has carried increasing attention in recent years. One of the main reasons is due to the fact that CRC patients with K-ras mutations will not benefit from anti-EGFR antibodies [15].

Percentage of K-ras mutations, which are known to work as an activated oncogene, occurs in 35%-45% of CRC worldwide [6,11,16]. Results from these studies are in agreement with our observation that ~40% of patients carry K-ras mutations. Few studies looked at the percentage of K-ras mutations in Saudi Arabia. For instance, a study reported that 32% of tumors (15/46) carry K-ras mutations [17]. Another study reported that 45% of tumors (97/221) carry K-ras mutations [18]. A third study reported that 56% of tumors (84/150) carry K-ras mutations [19]. It is not clear why there is a wide range difference in the percentage of K-ras mutations in Saudi Arabia and there is a clear need for a national survey that considers the possible reasons for these variations.

One of the main objectives of this study is to look into K-ras status and its correlation with patients’ characteristics as well as its association with clinicopathological features. We have found that the percentage of K-ras mutations occurrence was higher in young patients (i.e ≤50) and in the right-sided tumors. However, we found no significant association between the overall frequency of K-ras mutations and patients’ characteristics and/or clinicopathological features. In literature, conflicting results were documented regarding the association between K-ras mutations and patients’ characteristics and/or clinicopathological features [17-21]. For example, while some studies showed that females are significantly more likely to have mutant K-ras than males [18,22], other studies showed the opposite (i.e males are significantly more likely to have mutant K-ras than females) [17,23]. Further studies will be required to determine whether mutant K-ras is significantly correlated with CRC patients’ characteristics and clinicopathological features or not.

Delineating the pathogenetic importance of mutant K-ras in CRC is a successful step towards the control and management of this common type of cancer. In this study, 75% of K-ras mutations were found in codons 12 and 20% in codon 13. A similar observation was reported by others in which they found ~87% and ~14% of K-ras mutations in codons 12 and 13, respectively [17,19,24]. While lack of survival data is a limitation of our study, many studies showed that K-ras status did not significantly impact the clinical outcome and overall survival [11,17,18]. Due to the oncogenic nature of K-ras, we are currently following up on the patients included in this study for a longer period to correlate the survival data with K-ras mutations. We believe that it will be more informative to analyze survival data for each K-ras mutation in a large cohort. For example, results derived from studies that performed a comprehensive characterization of some commonly mutated oncogenes, such as p53 and anaplastic lymphoma kinase, concluded that prognostic significance of mutations is dependent on the region of the gene [25,26].

## Conclusions

In this small retrospective study, we showed that the rate of K-ras mutation in Saudi patients with CRC is 40% which is in agreement with most studies worldwide. K-ras mutation status did not significantly correlate with patients’ characteristics and clinicopathological features. Lastly, our data has suggested that further investigation would be required to establish the pathogenetic importance of K-ras mutations in larger cohorts as well as to address the correlation and its prognostic value. Furthermore, longer follow up to address the survival are warranted.
